# Removal of Parotid Tumor—Death from Cellulitis

**Published:** 1880-03

**Authors:** 


### REMOVAL OF PAROTID TUMOR—DEATH FROM CELLULITIS.

Dr. A. C. Post presented a tumor of the parotid gland, which he had taken from a patient sixty-one years of age. The tumor had been noticed for four months, and caused severe pain; to relieve this the operation was proposed. The mass was situated behind the sterno-cleido-mastoid muscle, and closely adherent to it. Much difficulty was encountered in its removal, and it was requisite to cut several branches of the cervical plexus of nerves. The wound healed by first intention, but in a few days opened, and suppuration occurred, which extended down below the clavicle. The cellulitis was of the gangrenous form. Death took place from exhaustion in eight days.

